# Blends of palm kernel oil, soybean oil and palm stearin as an alternative to milk fat for frozen dessert application

**DOI:** 10.1007/s13197-022-05507-z

**Published:** 2022-06-23

**Authors:** T. Hasan, Y. Y. Thoo, C. L. Chew, P. S. Kong, L. F. Siow

**Affiliations:** 1 School of Science, Monash University Malaysia, Jalan Lagoon Selatan, 47500 Bandar Sunway, Selangor Malaysia; 2 Department of Food Technology and Nutrition Science, Noakhali Science and Technology University, Noakhali, 3814 Bangladesh; 3 Sime Darby Plantation Research, R&D Centre - Carey Island, Lot 2664 Jalan Pulau Carey, 42960 Pulau Carey, Selangor Malaysia; 4 Monash-Industry Palm Oil Education and Research Platform (MIPO), Monash University Malaysia, Jalan Lagoon Selatan, 47500 Bandar Sunway, Selangor Malaysia

**Keywords:** Ternary blends, Milk fat, Palm, Solid fat content

## Abstract

In this study, the ternary blends of palm kernel oil (PKO), soybean oil (SBO) and two types of palm stearin (PS) (PS33 and PS38) were systematically prepared and evaluated for their application as an alternative to milk fat in frozen dessert. The physicochemical characteristics namely fatty acids constituent, triacylglycerols (TAGs) composition, melting behavior, solid fat content (SFC) and microstructure were studied. All ternary blends of PKO/SBO/PS33 and PKO/SBO/PS38 had comparable microstructure to that of milk fat. However, 80/15/5 (PKO/SBO/PS33) and 80/15/5 to 80/5/15 (PKO/SBO/PS38) mixtures were found to have similar SFC as milk fat at temperature above 20 °C, allowing these ternary blends to be completely melted at body temperature. With this, 80/15/5 (PKO/SBO/PS33) and 80/15/5 to 80/5/15 (PKO/SBO/PS38) mixtures were able to provide similar meltdown and mouth feel as milk fat at room temperature. Nonetheless, fatty acids constituent, TAGs composition and melting behavior of the blends were different from milk fat. This study showed that ternary blends of PKO, SBO and PS at ratios 80/15/5 (PKO/SBO/PS33) and 80/15/5 to 80/5/15 (PKO/SBO/PS38) can be potentially used as a milk fat alternative for frozen dessert application based on its similar microstructure and SFC profile as the milk fat.

**Supplementary Information:**

The online version contains supplementary material available at 10.1007/s13197-022-05507-z.

## Introduction

Frozen desserts are a group of aerated foods that are consumed in a frozen state, including ice creams, frozen yogurts, sorbets, mellorine, frozen custard, water ices and etc. (Goff and Hartel 2013). The most commonly used ingredients in a frozen dessert include milk solids (with or without milk fat), sugars/sweeteners, stabilizers, emulsifiers, different colors and flavors, and water. A typical frozen dessert (i.e., ice cream) may contain as low as 2–3% fat in low-fat products to as high as 16% fat in super-premium products (Clarke 2012; Goff and Hartel 2013). The fat constituent of frozen dairy desserts acts as a carrier and synergist for added flavor compounds, is responsible for good melting characteristics, and support in providing structure to the final products. As a result, fats impart some crucial functional and organoleptic attributes such as smooth and creamy texture, enhances the richness of flavor (especially milk fat) etc. (Alan et al. 2019; Motamedzadegan et al. 2020). In a frozen dairy dessert, fat usually comes from milk fat, such as cream, butter, and anhydrous milk fats. Milk fat has a unique fatty acids profile containing many short-chain, volatile fatty acids that govern much of its distinctive flavor and spare for a broad melting range (− 40–40 °C) (Ten Grotenhuis et al. 1999). This wide melting range contributes to proper melting of the fat crystals and collapsing of the foam structure (for aerated frozen desserts). These properties result in the mouth coating characteristics inside the mouth, leading to an increased perception of creaminess (Goff 2006; Clarke 2012). However, the frozen dairy desserts are off-limits to those who have metabolic diseases namely cow milk allergy. Besides, vegetarianism (except lactovegetarians and lacto­ovo vegetarians) restricts people from consuming milk and dairy products due to their practice of avoiding any animal source food in the diet (Silva et al. 2020). Moreover, milk fat is one of the costliest among all the edible fats while fats/oils of plant sources are generally much less expensive than milk fat (Goff and Hartel 2013). The price of butterfat (99%) is USD 4000–5000 per metric ton while the price of edible oils is USD 1000–2000 per metric ton (USDA 2021).

Consequently, in food industry, plant (vegetable) originated fats and oils are extensively used as an alternative choice of fat sources for many food preparations including frozen desserts. Palm kernel oil (PKO) is derived from the kernel of the oil palm (*Elaeis guineensis*) fruits. It has a melting range of 26–28 °C, which makes it a semi-solid fat at room temperature. PKO is a rich source of medium-chain fatty acids such as lauric and myristic acids (Fauzi et al. 2013; Biswas et al. 2016). It has a relatively sharp melting profile that makes it suitable for the production of confectioneries and other specialty fats. Soybean oil (SBO) is one of the most abundant and economic oils globally and also a rich source of ω–3 and ω–6 fatty acids. SBO has been reported to reduce the serum cholesterol and LDL levels, and consequently, contributing to averting the risk of cardiovascular diseases (CVDs) (Kummerow et al. 2007). Although SBO has wide functionality, poor plasticity, spreadability and liquid nature at room temperature limit its application in fat products. Palm stearin (PS) is a natural and cheaper solid fraction produced by fractionation of palm oil. PS melts at high temperature (44–56 °C) that makes it challenging to use PS directly in the manufacture of solid fat products. Because of its hard nature, it does not completely melt at body temperature and also it provides low plasticity to the final products. Hence, PS is often blended with lower melting oils to improve its melting properties (Fauzi et al. 2013; Biswas et al. 2016; Liu et al. 2018).

It is challenging to formulate frozen desserts with vegetable oils since vegetable oils have different physicochemical properties compared to that of milk fat. The specific distribution pattern of fatty acids on the glycerol backbone of fats and oils arises as the prime concern as it governs their physicochemical properties and application in natural state (Karabulut et al. 2004; Taghvaei and Jafari 2015; Alan et al. 2019). Therefore, a number of chemical processes (i.e., hydrogenation and interesterification) or physical processes (i.e., fractionation and blending) have been developed to modify the functional features of oils and fats (Hashempour-Baltork et al. 2016; Alan et al. 2019). Among these, blending is the most simple, easy and cost-effective process for fat modification. Blends of multiple vegetable oils could potentially alter the physicochemical properties of oils (Hashempour-Baltork et al. 2016) and hence, could alter the techno-functionality of the final product to provide desirable attributes such as mouth-feel and hardness (Liu et al. 2018; Motamedzadegan et al. 2020). Nonetheless, in some cases, there is a need for optimization of the blending to achieve a desired quality. Earlier study demonstrated that the overall quality of blended fats and oils is compromised due to the reduction of essential (i.e. ω–3 and ω–6) fatty acids (Hashempour-Baltork et al. 2016).

In the present study, we aim to identify suitable blends of plant-based oil as alternative to milk fat in frozen dessert (i.e., ice cream) application. The specific objective of this study was to assess the physical and chemical properties of binary blends of PKO/SBO by analyzing their fatty acids content, triacylglycerols (TAGs) composition, melting behavior, and solid fat content (SFC). Taking into consideration that SFC is regarded as the pivotal parameter for frozen dessert (i.e., ice cream) formulation, we primarily compared the SFC of PKO/SBO mixtures to that of milk fat. Based on the SFC of the binary mixtures, we systematically prepared ternary blends of PKO/SBO/PS and evaluated their solid fat content, as well as fatty acids profile, triacylglycerol constituents, and melting behavior for potential use as milk fat alternative for frozen dessert (i.e., ice cream) preparation.

## Materials and methods

### Materials

Refined, bleached, and deodorized (RBD) palm kernel oil (PKO) (Price: MYR 20/kg) and two different types of RBD palm stearin (PS) [IV 33 (PS33) and IV 38 (PS38)] (Price: MYR 30/kg) were received from Sime Darby Plantation, Malaysia. RBD soybean oil (SBO) (Price: MYR 9/kg) and unsalted cow milk butter (Anchor, New Zealand) containing ~ 83% fat (Price: MYR 57/kg) were purchased from a local supermarket at Bandar Sunway, Selangor, Malaysia. All the samples were stored at refrigerator (~ 4 °C) prior to use. Analytical or high-performance liquid chromatography (HPLC) grade chemicals and solvents (Sigma-Aldrich, USA) were used for all analyses.

### Milk fat preparation

Butter samples were completely melted and centrifuged at 6000 rpm for 10 min. The clear supernatant was carefully collected and conducted for fat analysis according to standard protocol (ISO 3727, part 1–3). The fat content was found to be > 99.9%.

### Blend preparation

PKO, SBO and PS were melted at 60 °C prior to use. The binary blends of PKO and SBO were formulated at concentrations from 0 to 100% (w/w) in 20% increments. The ternary mixtures of PKO, SBO and PS were prepared in the following ratios (w/w): PKO:SBO:PS = 80:15:5, PKO:SBO:PS = 80:10:10 and PKO:SBO:PS = 80:5:15. The blend compositions are provided in Supplementary Table S1. The samples were thoroughly mixed and then stored at room temperature until further analysis.

### Fatty acids

The fatty acids composition of the samples was analyzed using gas chromatograph (GC) (Perkin Elmer Clarus 500 GC, USA) equipped with an Elite-FFAP column (30 m × 0.32 mm i.d. × 0.25 μm) and a flame ionization detector (FID). About 50 mg of samples were dissolved in 500μL of heptane. The mixtures were then esterified by adding 1 mL of 1 N sodium methoxide at room temperature and mixed vigorously with a vortex mixer for 60 s. After separation of two distinct layers, the upper clear supernatant was collected as FAME. For GC analysis, an injection volume of 0.5 μL and a helium carrier gas at 1.0 mL/min flow rate was applied with the injector and detector temperatures set at 250 °C. The oven temperature was programed as follows: hold at 100 °C for 5 min, heat from 100 to 240 °C at 4 °C/min and hold for 15 min at 240 °C. The fatty acids were identified using respective fatty acid methyl esters (FAME) standards and an area percent normalization method was employed to quantify them.

### Triacylglycerols (TAGs)

TAGs profile of milk fat, PKO, SBO, PS and their blends was determined using mathematical algorithm as proposed by Filho and co-workers (Filho et al. 1995). According to the equations, if a, b and c are the molar percentages of fatty acids A, B and C, then the molar percentage of triacylglycerols containing only one acid such as the fatty acid A can be calculated as:

$$\% {\text{AAA}} = {\text{a}}^{3} /10000$$

Again, the molar percentage of triacylglycerols containing two acids such as A and B is expressed as:

$$\% {\text{AAB}} = 3 \times {\text{a}}^{2} {\text{b}}/10000$$

And the molar percentage of triacylglycerols containing three acids (A, B and C) is measured as:

$$\% {\text{ABC}} = 6 \times {\text{a}} \times {\text{b}} \times {\text{c}}/10000$$

This method showed high correlation between TAGs of several vegetable oils analysed by GC and those of the suggested formula. The aforementioned method generates a very large number of TAGs and hence, to reduce the number of components, all the TAGs with the same carbon number were added as a group (MacGibbon and Taylor 2006).

### Melting behavior

The melting properties of the blends were analyzed by using a differential scanning calorimetry (Pyris 4000 DSC; Perkin-Elmer, USA) according to the method of Biswas et al. (2016) with slight modifications. Nitrogen was purged at the flow rate of 20 mL/min and indium was used to carry out calibration of the instrument. About 5–10 mg of the melted samples were hermetically sealed in an aluminum pan and an empty pan was used as a reference. The samples were held isothermally at 80 °C for 2 min to destroy crystal memory, and then cooled to − 50 °C at a rate of 5 °C/min. The samples were then held isothermally for 2 min, before being heated to 80 °C at a rate of 5 °C/min. The melting thermograms were recorded from − 50 to 80 °C.

### Solid fat content (SFC)

The SFC of the samples was determined from their melting thermograms as described by Sung and Goff (2010). The SFC was calculated as a function of temperature from − 40 to 60 °C by the fraction of the total area contained under the melting curve that is passed at any given temperature.

### Microstructure

The microstructure of milk fat and ternary mixtures of PKO/SBO/PS was observed using a microscope (Olympus CX43, Tokyo, Japan) equipped with the polarized light imaging system following the method of Fauzi et al. (2013) with slight modifications. The samples were first heated at 60 °C for 30 min to eliminate crystal memory. About 10 μL of melted sample was placed on the microscopic slide which was pre-heated to the same temperature and carefully covered by a pre-heated cover slip. The slides were then stored in a temperature-controlled cabinet at 22 ± 1 °C for 48 h to ensure proper crystallization. The samples were observed under a digital camera (Nikon, DS-Filc, Tokoyo, Japan) and taken at 100 × magnification.

### Statistical analysis

All the measurements were performed in triplicates and the results were presented as mean ± standard deviation (SD). Differences among the samples were statistically analyzed using one-way ANOVA followed by Tukey’s test at level of *p* < 0.05. Regression models were used to analyze the straight-line relationships between SFC and saturated–unsaturated fatty composition of the oil blends. Statistical analyses were conducted using SPSS (v26.0) for Windows (SPSS Inc., Chicago, IL, USA).

## Results and discussion

### Cost of milk fat and oil blends

As the plant-based oils were mixed at different ratios, the cost of preparing 1 kg of the PKO/SBO/PS ternary blends ranging from MYR 19 to MYR 21. On the other hand, milk fat costed about MYR 57 per kg. Hence, the price of milk fat was much higher compared to all the ternary blends of PKO/SBO/PS.

### Fatty acids composition

The fatty acids composition of PS, PKO, SBO, and their binary blends are presented in Supplementary Table S1. Lauric acid (45.3%) was the predominant fatty acid in PKO, followed by oleic (17.4%), myristic (17.3%) and palmitic (9.5%) acids. SBO was mainly composed of long-chain fatty acids such as linoleic, oleic, palmitic, and linolenic acids in decreasing order. Both PS33 and PS38 were rich in palmitic (59.1% and 56.4%), oleic 27.0% and 29.3%), linoleic (5.6% and 6.0%) and stearic (5.2% and 4.9%) acids, respectively. These fatty acid profiles are consistent with those reported in the literature (Nor Hayati et al. 2009; Biswas et al. 2016; Alan et al. 2019). In PKO/SBO blends, the proportion of medium-chain fatty acids (lauric and myristic) decreased and that of long-chain fatty acids (i.e., palmitic, stearic, oleic, linoleic, and linolenic) increased gradually with the increment of SBO.

The fatty acid content of milk fat is shown in Table 1. It is reported that animal breeds (Sağdıç et al. 2004), the dietary practice of the animals (Mallia et al. 2008) and seasonal variations (Ledoux et al. 2005) affect the fatty acids profile of milk fat. In the current study, milk fat was found to contain a mixture of short-, medium- and long-chain fatty acids, however, the most abundant fatty acids include palmitic (30.2%), oleic (23.3%), stearic (14.9%) and myristic (13.8%) acids. The present findings are in line with previously published research (Givens and Shingfield 2006; Månsson 2008). Table 1 also represents the fatty acids composition of ternary mixtures of PKO/SBO/PS. When both PS33 and PS38 increased in the mixtures, the percentage of medium (lauric and myristic) and long-chain (palmitic and oleic) fatty acids increased with a gradual decrease in the concentration of long-chain polyunsaturated fatty acids (linoleic acids). The fatty acids composition of all the ternary blends was not comparable to that of milk fat.

**Table 1:** Fatty acid composition (% peak area) of milk fat, and PKO/SBO/PS mixtures

Blend	C_12_	C_14_	C_16_	C_18_	C_18:1_	C_18:2_	C_18:3_	Others
Milk fat	4.8 ± 0.1^a^	13.8 ± 0.5^a^	30.2 ± 4.9^a^	14.9 ± 1.8^a^	23.3 ± 2.5	1.7 ± 0.4^a^	0.7 ± 0.1^a^	10.5 ± 1.2
G (80:15:5)	33.8 ± 0.2^b^	12.4 ± 0.2^b^	14.4 ± 0.7^b^	3.2 ± 0.0^b^	19.5 ± 0.2	12.2 ± 0.3^b^	0.2 ± 0.0^b^	4.4 ± 0.4
H (80:10:10)	34.3 ± 1.8^b^	13.4 ± 0.4^a^	15.6 ± 0.2^b^	3.3 ± 0.3^b^	20.1 ± 1.2	8.7 ± 0.7^c^	0.2 ± 0.0^b^	4.4 ± 0.5
I (80:5:15)	34.9 ± 1.7^b^	13.5 ± 0.2^a^	16.2 ± 0.4^b^	4.2 ± 0.2^c^	20.7 ± 1.3	6.2 ± 0.6^d^	0.1 ± 0.0^c^	4.1 ± 0.8
J (80:15:5)	33.5 ± 0.1^b^	13.4 ± 0.1^a^	13.8 ± 0.1^b^	3.2 ± 0.0^b^	20.0 ± 0.1	10.8 ± 0.0^e^	0.2 ± 0.1^b^	5.1 ± 0.5
K (80:10:10)	34.3 ± 2.3^b^	13.4 ± 0.2^a^	15.6 ± 1.2^b^	3.2 ± 0.3^b^	20.1 ± 1.7	8.3 ± 0.5^c^	0.2 ± 0.0^b^	4.8 ± 0.5
L (80:5:15)	34.9 ± 0.4^b^	13.7 ± 0.2^a^	16.5 ± 0.7^b^	3.3 ± 0.1^b^	20.9 ± 0.1	6.0 ± 0.7^d^	0.1 ± 0.0^c^	4.6 ± 0.7

*PKO* Refined, bleached, and deodorized palm kernel oil, *PS* Refined, bleached, and deodorized palm stearin, *SBO* Refined, bleached, and deodorized soybean oil. Others include fatty acids like butyric, caproic, caprylic, capric, arachidic etc.

^a–e^Values with the different superscript letter within the same column are significantly (*p* < 0.05) different

### TAGs composition

TAGs composed of three fatty acids esterified to a glycerol moiety at stereospecific positions (MacGibbon and Taylor 2006). The properties of the TAG molecule are influenced by the fatty acid composition as well as their arrangement within the glycerol backbone (Jensen et al. 1991; Tzompa-Sosa et al. 2014). Supplementary Table S2 shows the TAG composition of PS, PKO, SBO, and their binary mixtures. PKO showed a wide variety of TAGs, mostly dominated by C36 to C42 due to the higher concentration of medium-chain fatty acids (i.e., lauric and myristic acids) (Supplementary Table S2). SBO was rich in TAGs mainly within the range of C50-C54 as the presence of long-chain fatty acids in SBO (i.e., palmitic, stearic, oleic, linoleic, and linolenic) was observed (Supplementary Table S2). PS (both IV 33 and IV 38) was predominated by TAGs ranging from C48 to C52 owing to the higher content of long-chain fatty acids (i.e. palmitic, stearic, oleic and linoleic) (Supplementary Table S2). In PKO and SBO blends, the content of high molecular weight TAGs (C52 to C54) increased with the increment of SBO in the mixture. For example, the proportion of C52 and C54 risen from 2.7 to 17.5% and 3.1 to 37.0%, respectively, in mixture B to E.

Milk fat displayed a broad range of TAGs, predominated by C46-C52 that is ranging from 11.0 to 15.4% (Table 2). This variation in TAGs content might be because of different types of fatty acids (i.e., short, medium and long-chain) present in milk fat (Table 1). This wide variety in TAG profile is mostly responsible for the complex thermal behavior and broad melting temperature (from − 40 to 40 °C) of milk fat (Shi et al. 2001; Mazzanti et al. 2004). Blending of PKO/SBO/PS showed a mixture of TAG constituents, mostly within C36-C48 (Table 2). This variety of TAGs is likely caused by the variations of fatty acids in the ternary mixture (Table 1). No significant (*p* > 0.05) difference was observed in TAG compositions within the two types of PS (IV 33 and IV 38). The TAGs composition of all ternary blends in this study is reasonably different from that of milk fat.

**Table 2:** TAG composition (%) of milk fat, and PKO/SBO/PS mixtures

Blend	C36	C38	C40	C42	C44	C46	C48	C50	C52	C54	Others
Milk fat	4.2 ± 0.9^a^	5.0 ± 1.0^a^	6.1 ± 0.5^a^	6.7 ± 0.2^a^	8.8 ± 0.1^a^	12.0 ± 0.2	14.8 ± 0.5^a^	15.4 ± 0.8^a^	11.0 ± 1.3^a^	4.5 ± 1.8^a^	11.6 ± 2.6
G (80:15:5)	8.4 ± 0.2^b^	9.4 ± 1.0^b^	11.2 ± 0.4^b^	18.9 ± 0.6^b^	12.6 ± 0.1^b^	11.7 ± 0.6	12.8 ± 1.0^a^	4.6 ± 0.2^b^	3.3 ± 0.5^b^	2.4 ± 0.2^b^	4.8 ± 0.6
H (80:10:10)	8.8 ± 0.6^b^	9.9 ± 0.8^b^	11.9 ± 0.6^b^	19.0 ± 1.2^b^	13.0 ± 0.3^b^	11.8 ± 0.8	11.7 ± 1.2^b^	4.4 ± 0.3^b^	3.0 ± 0.9^b^	1.8 ± 0.2^b^	4.7 ± 0.3
I (80:5:15)	8.9 ± 1.3^b^	10.1 ± 0.9^b^	12.3 ± 0.3^b^	19.0 ± 2.1^b^	13.1 ± 0.4^b^	11.9 ± 0.5	11.3 ± 0.7^b^	4.3 ± 0.3^b^	2.9 ± 0.8^b^	1.7 ± 0.2^b^	4.8 ± 0.3
J (80:15:5)	8.8 ± 1.5^b^	9.9 ± 1.1^b^	11.9 ± 0.7^b^	19.0 ± 1.4^b^	13.0 ± 0.4^b^	11.8 ± 0.3	11.7 ± 0.4^b^	4.4 ± 0.6^b^	2.9 ± 0.5^b^	1.8 ± 0.1^b^	4.7 ± 0.4
K (80:10:10)	8.6 ± 0.8^b^	9.8 ± 1.4^b^	11.8 ± 0.6^b^	18.9 ± 1.2^b^	13.1 ± 0.6^b^	11.9 ± 0.1	11.9 ± 0.5^b^	4.5 ± 0.4^b^	3.0 ± 0.2^b^	1.9 ± 0.1^b^	4.7 ± 0.4
L (80:5:15)	9.0 ± 0.6^b^	10.1 ± 1.2^b^	12.5 ± 0.7^b^	18.9 ± 1.0^b^	13.1 ± 0.8^b^	12.0 ± 0.1	11.0 ± 0.2^b^	4.3 ± 0.6^b^	2.8 ± 0.3^b^	1.6 ± 0.1^b^	4.8 ± 0.3

*PKO* Refined, bleached, and deodorized palm kernel oil, *PS* Refined, bleached, and deodorized palm stearin, *SBO* Refined, bleached, and deodorized soybean oil

^a–e^Values with the different superscript letter within the same column are significantly (*p* < 0.05) different

### Melting behavior

The melting curves of PS, PKO, SBO and their binary blends are illustrated in Supplementary Fig. S1. The single blend of PKO and SBO showed substantially different melting thermograms, which are possibly because of their difference in fatty acids content and TAGs composition. PKO exhibited an endotherm with a comparatively small temperature range of 15.4–30.1 °C, attributed to the higher content of short- and medium-chain saturated fatty acids (Supplementary Table S2). SBO showed a lower melting temperature (from − 29.2 to − 22.3 °C) compared to PKO, relating to the abundance of long-chain mono- and polyunsaturated fatty acids (Supplementary Table S2). Both PS33 and PS38 demonstrated two distinct endothermic peaks at 9 and 54 °C and 8 and 52 °C, respectively, with an exotherm around 10 to 25 °C between them. This exotherm might be due to the polymorphic transformation during the DSC melting (Tan and Man 2002). The oleic fraction might be responsible for the lower temperature endotherm (T_1_), while the higher temperature endotherm (T_2_) is probably because of the stearin fraction. These findings corroborate with several studies (Nor Hayati et al. 2009; Fauzi et al. 2013; Biswas et al. 2016). Mixture B to D of PKO/SBO exhibited two broad endothermic peaks, with T_1_ within the range of − 22.7–− 19.9 °C and T_2_ between 20.7 and 25.2 °C. Besides, mixture E of PKO/SBO displayed a major endothermic peak (T_1_) at about − 23.4 °C with one/two minor shoulder peaks.

**Fig. 1:**
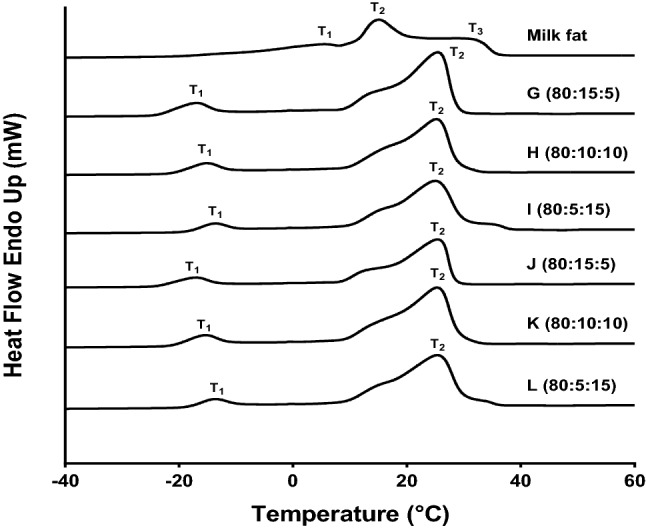
DSC melting thermograms of milk fat, and PKO/SBO/PS mixtures

For milk fat, a primary endothermic peak (T_2_) was noted at about 15.7 °C, with two secondary shoulder peaks, T_1_ at 5.8 °C and T_3_ at 30.2 °C (Fig. 1). This melting features of milk fat is comparable to those previously reported (Timms 1980; Ten Grotenhuis et al. 1999; Shen et al. 2001). Among the ternary mixtures of PKO/SBO/PS, very small differences in melting temperatures were noticed among blends containing the two types of PS. Blend G to L showed two separate endothermic peaks: one (T_1_) between − 17.4 and − 13.3 °C and the other (T_2_) ranging from 23.3 to 25.3 °C (Fig. 1). These differences are possibly due to the variation in fatty acid constituents and corresponding TAGs of PKO, SBO and PS. In the present study, the melting profile of the ternary blends of PKO/SBO/PS was different from those of milk fat.

### SFC profile

The SFC profile refers to the proportion of solid fraction present in a lipid at each temperature. It is an influential factor to many fundamental characteristics such as physical stability, organoleptic properties, and spreadability of fatty foods (dos Santos et al. 2014). Hence, the SFC is extensively applied to express and comprehend food properties and applications including its behavior in diverse storage, processing and consuming conditions. The SFC of PS33, PS38, PKO, SBO, and binary blends of PKO/SBO at temperatures ranging from 0 to 60 °C are shown in Supplementary Fig. S2. PKO exhibited a sharp melting profile and was completely melted at temperature above 35 °C, which could be due to the presence of high amount of medium-chain (i.e., lauric and myristic) saturated fatty acids (Supplementary Table S2). While SBO remains as liquid throughout the temperature range of 0 to 60 °C, which is presumably explained by its high content of long-chain mono (oleic) and polyunsaturated (linoleic) fatty acids (Supplementary Table S2). Both PS33 and PS38 displayed the maximum SFC at 25 °C and below, and completely liquified at temperature above 55 °C. These findings were supported by earlier studies conducted on PKO, SBO and PS (Nor Hayati et al. 2009; Fauzi et al. 2013; Biswas et al. 2016), in which PKO was melted at temperature 25 °C, SBO was readily liquid above 0 °C and PS had the highest SFC at low temperatures (below 25 °C). In PKO/SBO mixture (blend B to E), the SFC was noticed to reduce gradually with the increasing temperature, and had the highest decline between 15 and 25 °C. This behavior was attributed mainly to the large proportion of TAGs that melted within these temperature ranges.

**Fig. 2:**
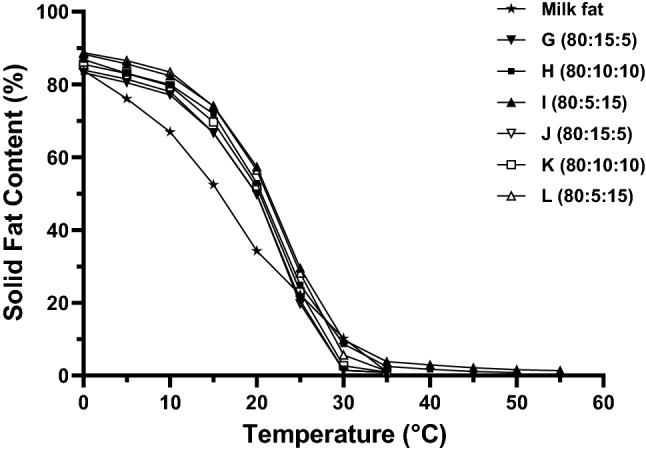
Solid fat content of milk fat and PKO/SBO/PS ternary mixtures

Milk fat exhibited around 22–34% solid fat at room temperatures (20–25 °C) with no solid fats detected at temperature above 35 °C (Fig. 2). This finding is in agreement with the earlier study by Shen and co-workers (Shen et al. 2001) where milk fat exhibited 17–30% SFC at room temperatures and 0% solids above the body temperature. In the present study, the SFC of blends B and C of PKO/SBO were the closest to that of milk fat compared to other blends from 0 to 20 °C. At temperature above 20 °C, the SFC of milk fat is higher than both blend B and C. Therefore, PS with higher SFC at 20 °C and above, was subsequently added (5–15%) into the blends and making the mixture.

SFC is the most crucial parameter to consider for frozen desserts such as ice cream, where it must be melted completely in the mouth to gain a good acceptability. The SFC of ternary blends (blend G to L) increased with the increasing PS in the mixture (Fig. 2). The SFC of mixture G to I (80/15/5 to 80/5/15 PKO/SBO/PS33) was found to be slightly different from those of J to L (80/15/5 to 80/5/15 PKO/SBO/PS38), which is presumably because of the difference in fatty acids concentration. Overall, the ternary blends of PKO/SBO/PS had a comparable SFC to that of milk fat, including at temperature above 20 °C. Majority of the ternary blends were also able to be melted completely within the body temperature (37 °C), except for blend H (80/10/10 PKO/SBO/PS33) and I (80/5/15 PKO/SBO/PS33) that had some solid fats remaining above 37 °C. At temperature 20 °C and below, the SFC value of all ternary mixtures were significantly higher (except for 0 °C) than milk fat. It has been reported that the SFC values between 20 and 38 °C indicate product stability at room temperature and influence the mouth feel induced by fat (dos Santos et al. 2014). Hence, frozen dessert (i.e., ice cream) prepared with blend G and blends J to L would have a similar meltdown and resembling mouth feel at room temperature compared to milk fat frozen dessert. In this regard, blend G and blends J to L could potentially be used as an alternative to milk fat for frozen dessert preparation.

Multiple regression analysis is useful in assessing the straight-line relationships among two or more variables. Hence, the influence of saturated–unsaturated fatty composition of the oil blends on SFC from − 5 to 5 °C (operational temperature of frozen dessert like ice cream) were analyzed using a multiple regression model, as described by Naeli et al. (2018):

$${\text{SFC}}_{{{\text{f}}\left( {{\text{SFA}},\;{\text{UFA}}} \right)}} = {\text{a}}\left( {{\text{SFA}}} \right) + {\text{b}}\left( {{\text{UFA}}} \right) + {\text{f}}$$

where SFA and UFA are saturated and unsaturated fatty acids content of oil blends, *a* and *b* are the regression coefficients and *f* is the random error.

Based on the regression analyses, several model equations were obtained as shown in Supplementary Table S3. All models showed excellent goodness of fit between predicted and experimental SFC values, having the correlation coefficients between predicted and experimental values of 1.000. The predicted and experimental SFC values from − 5 to 5 °C of all ternary blends of PKO/SBO/PS33 and PKO/SBO/PS38 are compared in Table 3. From the results, the proposed model could successfully predict the SFC content of the PKO/SBO/PS33 and PKO/SBO/PS38 mixtures which validated the use of multiple regression model using saturated–unsaturated fatty acids for predicting the SFC values. It was noted that, at any measured temperature, the SFC values increased with the increment of saturated fatty acids (SFA) in all the PKO/SBO/PS33 and PKO/SBO/PS38 mixtures. Furthermore, when compared with milk fat, all the PKO/SBO/PS33 and PKO/SBO/PS38 blends showed similar saturated–unsaturated fatty acids ratios and thus, comparable SFC values from − 5 to 5 °C (Table 3). Hence, it can be inferred that keeping a specific ratio of saturated–unsaturated fatty acids in a fat blend may result in a desirable solid fat content in the fat blend.

**Table 3:** Saturated–unsaturated fatty acid composition and predicted vs. experimental solid fat contents of milk fat, and PKO/SBO/PS mixtures

	SFA	UFA	SFC (%)					
			− 5°C_*Predicted*_	− 5°C_*Experimental*_	0°C_*Predicted*_	0°C_*Experimental*_	5°C_*Predicted*_	5°C_*Experimental*_
Milk fat	71.90	28.10	–	89.42	–	83.82	–	76.08
PKO/SBO/PS33								
G (80:15:5)	68.10	31.90	85.68	85.68	83.24	83.23	80.53	80.52
H (80:10:10)	71.00	29.00	88.44	88.44	86.88	86.87	83.05	83.04
I (80:5:15)	73.00	27.00	90.72	90.72	88.29	88.28	85.64	85.63
PKO/SBO/PS38								
J (80:15:5)	69.05	30.95	86.21	86.22	83.93	83.95	81.42	81.45
K (80:10:10)	71.37	28.63	87.79	87.80	85.48	85.50	82.93	82.97
L (80:5:15)	73.07	26.93	90.80	90.81	88.72	88.74	86.49	86.53

*PKO* Refined, bleached, and deodorized palm kernel oil, *PS33* Refined, bleached, and deodorized palm stearin with iodine value 33, *PS38* Refined, bleached, and deodorized palm stearin with iodine value 38, *SBO* Refined, bleached, and deodorized soybean oil, *SFA* Saturated fatty acids, *SFC* Solid fat content, *UFA* Unsaturated fatty acids

### Microstructure

**Fig. 3:**
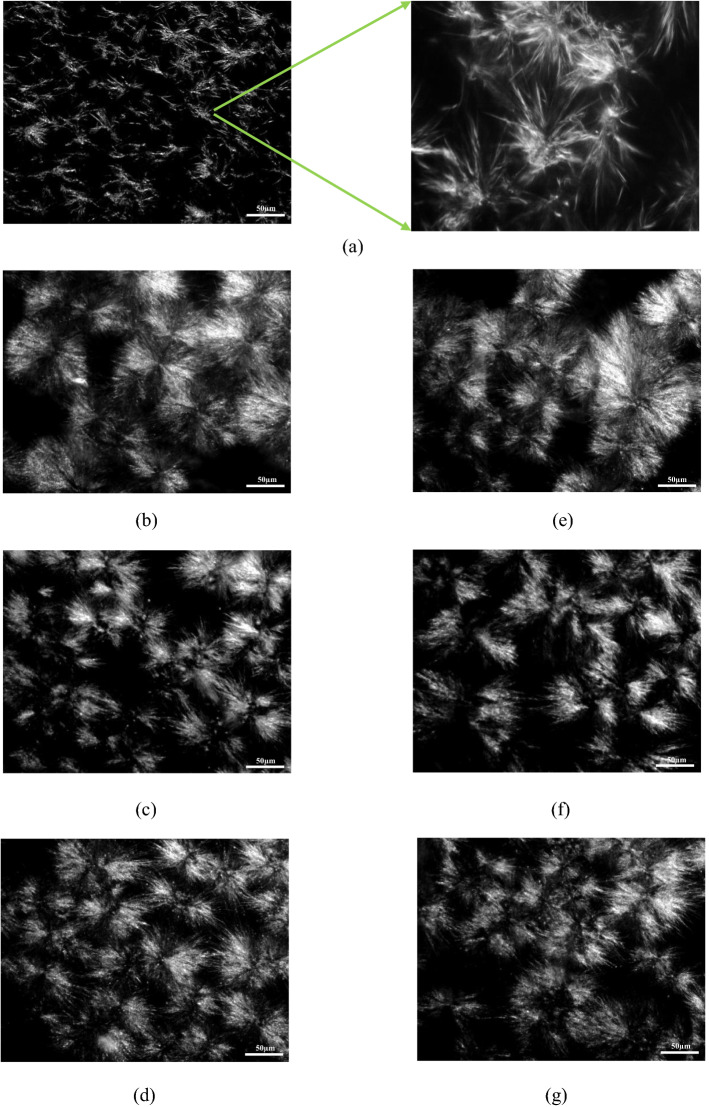
Polarized light microphotographs (100 × magnification) of a. milk fat, b. Blend G (80/15/5), c. Blend H (80/10/10), d. Blend I (80/5/15), e. Blend J (80/15/5), f. Blend K (80/10/10), g. Blend L (80/5/15)

The microstructure of crystal network of milk fat and ternary blends of PKO/SBO/PS were displayed in Fig. 3. Milk fat showed long, needle-like crystals of radially orientated type A spherulites (Fig. 3a). Similar behavior was also observed by Viriato et al. (2020), who reported needle-like microstructure of anhydrous milk fat. The polarized light micrographs of all ternary blends were found to be a mixture of tightly packed crystalline material (Fig. 3b–g). The crystal network morphologies of blend G to I (80/15/5 to 80/5/15 PKO/SBO/PS33) was found to be similar to those of blend J to L (80/15/5 to 80/5/15 PKO/SBO/PS38), which could be related to the similarity in their fatty acids composition (Table 1) and TAG species (Table 2). All ternary blends of PKO/SBO/PS exhibited spherulites consisting of needle-like crystals radiating and branching outward from the central nuclei. In the present study, all ternary blends of PKO/SBO/PS33 and PKO/SBO/PS38 were noticed to have comparable microstructure to that of milk fat.

## Conclusion

In the present study, all ternary blends of PKO/SBO/PS33 and PKO/SBO/PS38 had similar microstructure as milk fat. However, PKO/SBO/PS33 (80/15/5) and PKO/SBO/PS38 (80/15/5 to 80/5/15) demonstrated SFC profile comparable to that of milk fat, notably above 20 °C, giving similar meltdown to milk fat and hence, could be viable alternatives to milk fat for frozen dessert (i.e., ice cream) application. These findings will serve as a valuable reference for the food industries to aid in the selection of appropriate fat blends for nondairy-fat frozen desserts (i.e., ice cream) preparation.

## Supplementary Information

Below is the link to the electronic supplementary material.

Supplementary file1 (DOCX 129 KB)

## Data Availability

All data generated or analysed during this study are included in this published article.

## Abbreviations

PKO: Palm kernel oil
SBO: Soybean oil
PS: Palm stearin
SFC: Solid fat content
TAGs: Triacylglycerols
PS33: Palm stearin (IV 33)
PS38: Palm stearin (IV 38)
CVDs: Cardiovascular diseases
RBD: Refined, bleached, and deodorized
HPLC: High-performance liquid chromatography
GC: Gas chromatograph
FID: Flame ionization detector
FAME: Fatty acid methyl esters
IV: Iodine value
SD: Standard deviation
