# Supplementary material for: Blends of palm kernel oil, soybean oil and palm stearin as an alternative to milk fat for frozen dessert application
Source: J Food Sci Technol. 2022 Jun 23;59(8):3010–9. doi: 10.1007/s13197-022-05507-z (PMC9304505; doi:10.1007/s13197-022-05507-z)
Supplement: Supplementary file 1 — Supplementary file1 (DOCX 129 KB) [file 13197_2022_5507_MOESM1_ESM.docx]

**Table S1** Blends of PKO, SBO and PS (w/w).

| **Blend** | **PKO (%)** | **SBO (%)** | **PS IV 33 (%)** | **PS IV 38 (%)** |
| --- | --- | --- | --- | --- |
| Binary |  |  |  |  |
| A | 100 | 0 |  |  |
| B | 80 | 20 |  |  |
| C | 60 | 40 |  |  |
| D | 40 | 60 |  |  |
| E | 20 | 80 |  |  |
| F | 0 | 100 |  |  |
|  |  |  |  |  |
| Ternary |  |  |  |  |
| G | 80 | 15 | 5 |  |
| H | 80 | 10 | 10 |  |
| I | 80 | 5 | 15 |  |
| J | 80 | 15 |  | 5 |
| K | 80 | 10 |  | 10 |
| L | 80 | 5 |  | 15 |

*IV* Iodine value, *PKO* Refined, bleached, and deodorized palm kernel oil, *PS* Refined, bleached, and deodorized palm stearin, *SBO* Refined, bleached, and deodorized soybean oil.

**Table S2** Fatty acid composition (% peak area) of PS, PKO, SBO and their binary mixtures.

| **Blend** | **C_12_** | **C_14_** | **C_16_** | **C_18_** | **C_18:1_** | **C_18:2_** | **C_18:3_** | **Others** |
| --- | --- | --- | --- | --- | --- | --- | --- | --- |
| A (100:0) | 45.3±1.7^a^ | 17.3±0.2^a^ | 9.5±0.4^a^ | 2.5±0.2^a^ | 17.4±1.3^a^ | 2.9±0.4^a^ | ND | 5.0±0.4 |
| B (80:20) | 36.6±0.7^b^ | 13.6±0.1^b^ | 9.7±0.1^a^ | 2.9±0.1^a^ | 18.5±0.4^a^ | 13.2±0.3^b^ | 1.2±0.0^a^ | 4.5±0.2 |
| C (60:40) | 26.8±1.4^c^ | 10.0±0.2^c^ | 10.2±0.3^a^ | 3.4±0.2^b^ | 20.1±0.5^b^ | 24.0±0.9^c^ | 2.4±0.1^b^ | 3.3±0.4 |
| D (40:60) | 18.0±1.2^d^ | 6.7±0.2^d^ | 10.5±0.2^a^ | 3.7±0.2^b^ | 21.2±0.6^bf^ | 34.0±0.8^d^ | 3.6±0.0^c^ | 2.4±0.3 |
| E (20:80) | 8.6±0.5^e^ | 3.3±0.1^e^ | 10.9±0.1^a^ | 4.2±0.1^c^ | 22.6±0.5^cf^ | 44.4±0.6^e^ | 4.8±0.1^d^ | 1.2±0.1 |
| F (0:100) | ND | 0.1±0.0^f^ | 11.1±0.2^a^ | 4.7±0.1^d^ | 23.1±0.7^c^ | 54.3±0.4^f^ | 5.9±0.0^e^ | 0.7±0.1 |
| PS (IV 33) | 1.5±0.9^f^ | 1.7±0.5^g^ | 59.1±1.5^b^ | 5.2±0.0^e^ | 27.0±0.0^d^ | 5.6±0.2^g^ | 0.4±0.0^f^ | 0.1±0.0 |
| PS (IV 38) | 1.3±0.8^f^ | 1.8±0.3^g^ | 56.4±0.8^b^ | 4.9±0.1^d^ | 29.3±0.3^e^ | 6.0±0.0^g^ | 0.3±0.0^g^ | 0.1±0.0 |

*IV* Iodine value, *ND* Not detected, *PKO* Refined, bleached, and deodorized palm kernel oil, *PS* Refined, bleached, and deodorized palm stearin, *SBO* Refined, bleached, and deodorized soybean oil. Others include fatty acids like caproic, caprylic, capric, arachidic etc. ^a–g^Values with the different superscript letter within the same column are significantly (p<0.05) different.

**Table S2** TAG composition (%) of PS, PKO, SBO and their binary mixtures.

| **Blend** | **C36** | **C38** | **C40** | **C42** | **C44** | **C46** | **C48** | **C50** | **C52** | **C54** | **Others** |
| --- | --- | --- | --- | --- | --- | --- | --- | --- | --- | --- | --- |
| A (100:0) | 15.6±1.3^a^ | 16.0±0.9^a^ | 13.2±0.2^a^ | 19.0±0.5^a^ | 11.3±0.7^a^ | 6.6±0.7^a^ | 6.4±0.9^a^ | 2.0±0.3^a^ | 0.8±0.2^a^ | 0.6±0.1^a^ | 8.5±1.2 |
| B (80:20) | 9.0±1.6^b^ | 9.8±1.4^b^ | 9.6±0.8^b^ | 19.6±0.6^a^ | 13.1±0.4^b^ | 9.7±0.8^b^ | 14.2±1.9^b^ | 5.0±1.0^b^ | 2.7±0.7^b^ | 3.1±1.0^b^ | 4.5±1.4 |
| C (60:40) | 4.6±0.6^c^ | 6.0 ±0.6^c^ | 6.8±0.5^c^ | 16.7±0.8^b^ | 11.8±0.1^a^ | 10.9±0.3^c^ | 20.4±0.8^c^ | 7.1±0.5^c^ | 5.3±0.6^c^ | 7.9±0.9^c^ | 2.6±0.5 |
| D (40:60) | 1.8±0.3^d^ | 2.8±0.5^d^ | 3.7±0.4^d^ | 10.8±0.9^c^ | 8.7±0.5^c^ | 10.5±0.1^c^ | 24.0±0.1^d^ | 8.9±0.4^d^ | 9.8±0.8^d^ | 17.7±1.6^d^ | 1.1±0.3 |
| E (20:80) | 0.3±0.0^e^ | 0.6±0.1^e^ | 1.0±0.1^e^ | 3.6±0.3^d^ | 3.9±0.3^d^ | 7.1±0.3^d^ | 19.7±0.6^c^ | 8.8±0.1^d^ | 17.5±0.5^e^ | 37.0±1.3^e^ | 0.5±0.0 |
| F (0:100) | ND | ND | ND | ND | ND | ND | 0.2±0.0^e^ | 4.0±0.1^e^ | 27.6±0.3^f^ | 66.6±0.5^f^ | 1.6±0.2 |
| PS (IV 33) | ND | ND | 0.1±0.0^f^ | 0.1±0.1^e^ | 1.5±1.3^e^ | 3.8±2.0^e^ | 25.8±0.8^d^ | 40.7±2.0^f^ | 23.5±0.7^g^ | 4.6±0.0^g^ | ND |
| PS (IV 38) | ND | ND | 0.1±0.1^f^ | 0.2±0.1^e^ | 1.9±1.1^e^ | 4.3±1.7^e^ | 23.3±1.0^d^ | 39.6±1.6^f^ | 25.3±1.0^g^ | 5.5±0.2^h^ | ND |

*IV* Iodine value, *ND* Not detected, *PKO* Refined, bleached, and deodorized palm kernel oil, *PS* Refined, bleached, and deodorized palm stearin, *SBO* Refined, bleached, and deodorized soybean oil. ^a–h^Values with the different superscript letter within the same column are significantly (p<0.05) different.

**Table S3** Multiple linear regression models describing the solid fat content as a function of saturated-unsaturated fatty acid composition and goodness of fit of models.

| **Blend** | **Model equation** | **Goodness of fit** | | |
| --- | --- | --- | --- | --- |
|  |  | **r** | **R^2^** | **SE** |
| PKO:SBO:PS33 | SFC*_-5°C_* = (1.4×SFA) + (0.4×UFA) + (-16.4) | 1.000 | 1.000 | 0.000 |
|  | SFC*_0°C_* = (-4.091×SFA) + (-5.205×UFA) + (510.281) | 1.000 | 1.000 | 0.000 |
|  | SFC*_5°C_* = (3.168×SFA) + (2.190×UFA) + (-191.448) | 1.000 | 1.000 | 0.000 |
|  |  |  |  |  |
| PKO:SBO:PS38 | SFC*_-5°C_* = (-12.201×SFA) + (-14.618×UFA) + (1318.522) | 1.000 | 1.000 | 0.000 |
|  | SFC*_0°C_* = (-13.861×SFA) + (-16.501×UFA) + (1480.630) | 1.000 | 1.000 | 0.000 |
|  | SFC*_5°C_* = (-16.114×SFA) + (-19.061×UFA) + (1701.360) | 1.000 | 1.000 | 0.000 |

*PKO* Refined, bleached, and deodorized palm kernel oil, *PS33* Refined, bleached, and deodorized palm stearin with iodine value 33, *PS38* Refined, bleached, and deodorized palm stearin with iodine value 38, *SBO* Refined, bleached, and deodorized soybean oil, *SE* Standard error, *SFA* Saturated fatty acids (sum of C_12_, C_14_, C_16_ and C_18_), *SFC* Solid fat content, *UFA* Unsaturated fatty acids (sum of C_18:1_, C_18:2_ and C_18:3_), *r* Correlation coefficient between predicted and experimental values, *R^2^* Coefficient of determination.


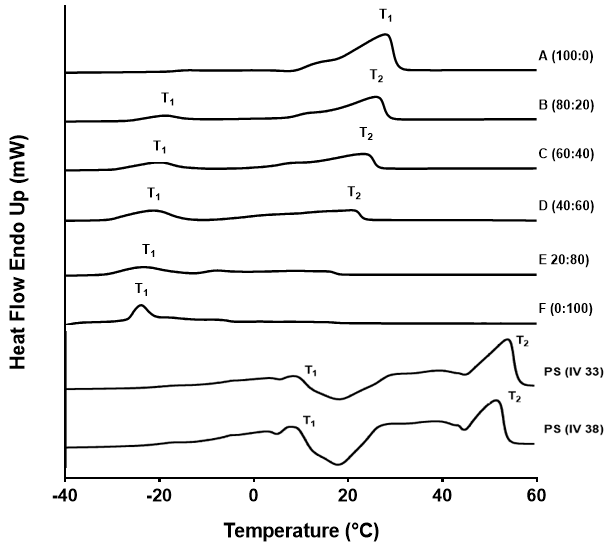


**Fig. S1** DSC melting thermograms of PS, PKO, SBO and their binary mixtures.

**Fig. S2** Solid fat content of PS, PKO, SBO and their binary mixtures.
